# Could a short training intervention modify opinions about mental illness? A case study on French health professionals

**DOI:** 10.1186/s12888-017-1296-0

**Published:** 2017-04-08

**Authors:** Murielle Villani, Viviane Kovess - Masfety

**Affiliations:** 1 Fondation Pierre Deniker, 36 avenue Raymond Poincaré, Paris, France; 2 Laboratoire de Psychopathologie et Processus de Santé, Paris Descartes University, Paris, EA4057 France; 3 Ecole des Hautes Etudes en Santé Publique, Paris, France

**Keywords:** Mental illness, Stigma, Attitudes, Targeted intervention, Health services professionals

## Abstract

**Background:**

In France, negative views on schizophrenia are pervasive, even among health professionals. Prior research suggests that the level of prejudice is lower when the illness is described with the example of a specific individual. This finding highlights the importance of designing local, targeted destigmatization campaigns. The present study aims to evaluate the benefits of a short intervention offering contact with psychiatric services users on reducing the stigma about mentally ill people, among a sample of Health Administrators and Students.

**Methods:**

Data were collected before (Time 0) and after (Time 1) a short training intervention program proposed to a sample of 121 Health Services Administrators and Students. This four-day workshop explained the multiple causes of mental illness, the clinical implications of psychosis and various mental disorders, the subjective experience of mental illness and the legal evolutions of users’ rights. The intervention was strongly based on live testimonies from users. Using a French version of the *Attitudes to Mental Illness* scale, we compared attitudes before and after the training intervention among 58 trainees having answered our questionnaire at Time 0 and Time 1.

**Results:**

After the training, a significantly lower endorsement of stigmatizing statements compared to baseline was found in one third (9 out of 27) of the items. These results plead for further research about the potential benefits of initiatives like this short intervention program on significantly reducing stigmatizing attitudes towards mentally ill people among Health Administrators and Students.

**Conclusions:**

The present study highlights the importance of further studying the effect of targeted interventions that offer first hand contact with persons with mental illness.

**Electronic supplementary material:**

The online version of this article (doi:10.1186/s12888-017-1296-0) contains supplementary material, which is available to authorized users.

## Background

Prejudice and discrimination towards people suffering from mental illness are common and socially damaging [1, 2]. This stigma contributes to negative outcomes, self-stigmatization and low self-esteem among persons with mental illness [3, 4]. However, attitudes towards mental illness can be modified over time by shifts in social perceptions or through media campaigns focused on mental health literacy about a disorder [5–8]. In France as in a number of other countries, negative views on schizophrenia are pervasive. Indeed, while the French are generally more familiar with the names of psychiatric disorders, as compared to Australians or Scots, there remains a clear lack of knowledge about mental illness as well as negative stereotypes about severe mental disorders including psychosis, and in particular schizophrenia [9]. That being said, in a large study on knowledge and attitudes toward severe mental disorders, when descriptors related to a person with a mental illness, prejudice was less overt [9]. This finding led to the conclusion that using personal testimonies and raising the visibility of persons with mental disorders in the community is likely to be more effective than generic campaigns in reducing stigma [9].

There have been several studies evaluating the effects of destigmatization campaigns. A recent literature review established that generic one-time campaigns have yielded limited results [10]. Studies quoted in the latter literature review have established that, in order to be efficient, interventions should focus on specific targets, include a follow-up over time, and include contact with psychiatric services users and their families [10]. In addition, the goals of such an intervention should be determined as a function of real users’ experience, and be accompanied by tangible evolutions in professional practice and social insertion of mentally ill people [10].

Literature reviews confirm the effectiveness of contact-based strategies for stigma reduction, in the general population as well as among health professionals [11–13]. For instance, contact-based education included in pharmacist students’ curricula in the form of social interactions with a person suffering from a mental illness, has proven to be effective in reducing mental health related stigma [14].

The present paper reports on a short training intervention aimed at exploring the modifications of attitudes towards people suffering from severe mental illness. The intervention was provided in the curriculum of the French National School of Public Health where public health services administrators are mandatorily trained. It was hypothesized that such training integrated into their curriculum as initial or continuing education, based on adapted scientific and clinical information together with life testimonies of users, would increase acceptance towards persons with mental illness. In addition, it was hypothesized that these health services professionals may help improve access to care and participate in the evolution of attitudes in the general population, resulting in a better integration of users into society. Indeed, they could play a strategic role in the delivery of care and in developing non-institutionalized care.

This shift was evaluated using the *Attitudes to Mental Illness* scale, the instrument used in the National United-Kingdom opinion surveys. We compare the attitudes and opinions of our sample before and after the intervention.

## Methods

### The short training intervention

The intervention was a four-day workshop focused on fundamental notions in psychiatry hosted at the school. It was designed to improve attendees’ knowledge about the multiple causes of mental illness, the clinical implications of psychosis and various mental disorders at all ages (schizophrenia, bipolar disorder, anxiety disorders, depression, eating disorders, addictions to alcohol and drugs, autism, behavior problems, perversions), the subjective experience of mental illness, and the legal evolution of users’ rights.

The workshop was not only designed and delivered by psychiatrists working in the field, but was also based on testimonies of psychiatric services users about their lives. These consumers had been contacted through users and families associations (Unafam, Profamille, Société française d’Alcoologie), had chosen to testify voluntarily, and were present in person on the training session location. They suffered from various mental disorders, in particular schizophrenia, bipolar disorders, anorexia or alcoholism. Users present to share their experience were free to organize their talk as they pleased and were compensated for their time. Testimonies lasted 30 min each, followed by a session with an opportunity for attendees to ask questions. Users shared their experiences regarding the consequences of their illness, their feelings about their symptoms and about psychiatric services. The sharing of personal experience represented the specificity of the training, in the hope that this would help modify perceptions of mentally ill persons and help better understanding them as individuals, as opposed to abstract representations that are often linked with dangerousness and weirdness. In practice, the sessions were a real discovery for most of the students, and always a moving moment for attendees as well as for speakers.

In addition, a film club was organized with a debate following the projection of a large audience movie presenting a character suffering from severe mental illness.

### Sample

The sample consisted of 121 Public Health Services administrators having attended an optional short training program about fundamental notions in psychiatry, in the context of their curriculum at the Ecole des Hautes Etudes en Santé Publique (EHESP), the National French school for Studies in Public Health. The school is a public institution, which provides training and conducts research in the field of public health.

Among the 121 eligible participants, 70 attended the training program in 2012 and 51 in 2014; 91 were women and 30 were men, and 90 were Students in Initial Training and 31 were Health Services Professionals (Managers or Administrators) in Continuing Education.

Overall, our sample consisted of 58 people having participated to the study before (Time 0) and after (Time 1) the training: 10 men and 48 women. Among them, 48 were students in Initial Training and 10 were Health Services Professionals (Managers or Administrators) in Continuing Education.

Time 0 assessments took place at the time of pre-registration for the workshop (2 to 3 weeks before the workshop), and Time 1 took place on average 8 days after the workshop, within a maximum of 30 days.

All respondents had volunteered for the optional training program in the context of their curriculum, and provided verbal informed consent for participation in this study. The study was carried out in compliance with the Helsinki declaration, and the rules in application in France and at the school. The program itself was approved by the Education Council and the Administrative Council of EHESP. Anonymity of all individual results has been respected all along the procedure.

### Instrument

The *Attitudes to Mental Illness* scale was designed to survey annually the attitudes towards mental illness among adults in England and Scotland at an initiative of the Department of Health. The questionnaire consists of 26 items derived from the *Community Attitudes Toward the Mentally Ill* (CAMI) scale [15]. The latter scale itself derives from the *Opinions about Mental Illness* [16] survey, with a greater focus on attitudes concerning the acceptance of mentally ill persons and/or mental health facilities in the community. The *Community Attitudes Toward the Mentally Ill* (CAMI) has shown good validity and demonstrated its usefulness as explanatory and predictive variables for studying attitudes in the community [15]. For the development of the *Attitudes to Mental Illness* questionnaire, the number of items was reduced to 26 and a 27th item about employment was added in 2000.

The questionnaire consists of a series of statements expressing a similar number of positive and negative views about mental illness and related to areas such as perceptions of mental illness, social distance from mentally ill people, the responsibility of society towards the mentally ill, the role of such people in society and the treatments for mental illness [17].

For analysis, the statements are generally categorized in four dimensions:

- Fear and exclusion of mental illness
- Integration of the mentally ill into the community
- Understanding and tolerance towards the mentally ill
- Causes of mental illness and needs for specialized services [18].

In the present study, the 27 items of the *Attitudes to Mental Illness* scale were translated into French by a bilingual translator, back translated, and adapted to French culture (Additional file 1). Respondents were asked to what extent they agreed or disagreed with each statement, with responses ranging from 1 (“*strongly Disagree*”) to 4 (“*strongly Agree*”). Since we aimed to measure the reduction of stigma between Time 0 (before the training) and Time 1 (after the training), the scoring of 14 statements was reversed.

The *Community Attitudes Toward the Mentally Ill* (CAMI), from which the *Attitudes to Mental Illness* scale derives, has been partially validated on a French-speaking sample, through one of its subscales, the *Community Mental Health Ideology* scale [19]. Also, some items of the *Attitudes to Mental Illness* scale correspond to similar items that have been validated previously for the purpose of a vignette-based population survey about attitudes and beliefs of the general French population about schizophrenia and major depression, conducted in 2013 [20]. Without being formally validated on a French sample, the *Attitudes to Mental Illness* scale has previously shown good psychometric qualities for some of its subscales/items on French and French-speaking samples. Also, the French version of the scale in the present sample showed good internal consistency with a Cronbach Alpha of .824.

### Data analysis

Data was analyzed using IBM SPSS Statistics 20 (Statistical Package for the Social Sciences, SPSS Inc., Chicago, IL). Missing values, representing less than 1,9% of the responses, were replaced using the Expectation Maximization procedure. A Shapiro-Wilk test showed that some items had highly skewed data distributions. In order to address this issue, a non-parametric test, the Wilcoxon signed-rank test for ordinal data in paired samples, has been used. The Wilcoxon test is frequently used to investigate pre-post scores in a sample of the same participants and is well adapted to skewed distributions. An alpha level of .05 was used to determine the significance of the results.

Mean scores to each item were used to perform data analysis, as it is the way the *Attitudes to Mental Illness* scale has been used in longitudinal researches in other European countries, which offers data for reference [17].

As mentioned in the previous section, the scoring of 14 statements has been reversed in order to align with a systematical stigmatizing trend. The items to which we have applied a reverse scoring are designated in Table 1 (Results).

**Table 1:** Mean scores at the AMI scale at Time 0 and Time 1

Item number	Scoring	Item wording	Dimension	Baseline value	Value difference T1-T0	Significance of difference
6	Reversed	Mental hospitals are an outdated means of treating people with mental illness	Integration of the mentally ill into the community	2,79	- 0,27	.011
25		It is frightening to think of people with mental problems living in residential neighborhoods	Fear and exclusion towards mental illness	2,70	- 0,07	.618
5	Reversed	Less emphasis should be placed on protecting the public from people with mental illness	Integration of the mentally ill into the community	2,49	- 0,54	.000
21	Reversed	Most women who were once patients in a mental hospital can be trusted as babysitters	Integration of the mentally ill into the community	2,40	- 012	.174
4	Reversed	Mental illness is an illness like any other	Integration of the mentally ill into the community	2,34	- 0,40	.007
26		Locating mental health facilities in a residential area downgrades the neighborhood	Fear and exclusion towards mental illness	2,11	- 0,25	.052
20	Reversed	People with mental illness are far less of a danger than most people suppose	Integration of the mentally ill into the community	1,89	- 0,49	.000
16		A woman would be foolish to marry a man who has suffered from mental illness, even though he seems fully recovered	Fear and exclusion towards mental illness	1,82	- 0,21	.015
17		I would not want to live next door to someone who has been mentally ill	Fear and exclusion towards mental illness	1,80	- 0,33	.002
15		People with mental illness should not be given any responsibility	Fear and exclusion towards mental illness	1,76	- 0,12	.314
2		There is something about people with mental illness that makes it easy to tell them from normal people	Causes of mental illness and needs for special services	1,76	+0,07	.437
27	Reversed	People with mental health problems should have the same rights to a job as anyone else	Integration of the mentally ill into the community	1,74	- 0,34	.001
14		There are sufficient existing services for people with mental illness	Causes of mental illness and needs for special services	1,72	- 0,07	.562
24	Reversed	Residents have nothing to fear from people coming into their neighborhood to obtain mental health services	Integration of the mentally ill into the community	1,71	- 0,12	.104
8	Reversed	People with mental illness have for too long been the subject of ridicule	Understanding and tolerance towards the mentally ill	1,69	- 0,17	.057
22	Reversed	The best therapy for many people with mental illness is to be part of a normal community	Integration of the mentally ill into the community	1,68	- 0,16	.070
9	Reversed	We need to adopt a far more tolerant attitude toward people with mental illness in our society	Understanding and tolerance towards the mentally ill	1,60	- 0,32	.000
18		Anyone with a history of mental problems should be excluded from taking public office	Fear and exclusion towards mental illness	1,56	- 0,14	.114
19	Reversed	No one has the right to exclude people with mental illness from their neighborhood	Integration of the mentally ill into the community	1,48	0,00	.840
7	Reversed	Virtually anyone can become mentally ill	Understanding and tolerance towards the mentally ill	1,47	- 0,04	.744
23	Reversed	As far as possible, mental health services should be provided through community based facilities	Understanding and tolerance towards the mentally ill	1,46	- 0,15	.129
3		As soon as a person shows signs of mental disturbance, he should be hospitalized	Fear and exclusion towards mental illness	1,40	- 0,12	.122
11		People with mental illness don’t deserve our sympathy	Understanding and tolerance towards the mentally ill	1,37	+ 0,02	.633
12		People with mental illness are a burden on society	Fear and exclusion towards mental illness	1,35	- 0,08	.310
13		Increased spending on mental health services is a waste of money	Understanding and tolerance towards the mentally ill	1,33	- 0,10	.246
1		One of the main causes of mental illness is a lack of self-discipline and will-power	Causes of mental illness and needs for special services	1,19	- 0,15	.004
10	Reversed	We have a responsibility to provide the best possible care for people with mental illness in our society	Understanding and tolerance towards the mentally ill	1,18	0,00	1

## Results

### Reduction of stigma between T0 and T1

We identified a significant reduction of stigma between Time 0 (before the training) and Time 1 (after the training), among the 58 respondents. A reduction of stigma equals to a decrease in the mean scores, which corresponds to a decrease in the level of agreement with stigmatizing statements. More precisely, one third of the statements (9 out of 27) - that is to say 5 statements of the Dimension “*Integration*”, 2 statements of the Dimension “*Fear and exclusion*”, one statement of the Dimension “*Understanding and tolerance*”, and one statement of the Dimension “*Causes of mental illness and needs for special services*” - showed a significant or very significant reduction of stigma.

As shown in Table 1, within the theme « *Integration of the mentally ill into the community* », 4 statements showed a very significant reduction of stigma (*p* < .01) and one statement a significant reduction of stigma (*p* < .05). The 4 statements with the most significant changes were the following:

- *“Less emphasis should be placed on protecting the public from people with mental illness”* (reversed)
- *“Mental illness is an illness like any other”*
- *“People with mental illness are far less of a danger than most people suppose”*
- *“People with mental health problems should have the same rights to a job as anyone else”.*

The statement “*Mental hospitals are an outdated means of treating people with mental illness*” has seen its level of agreement decrease significantly (*p* = .011). Although not significant, the decrease of agreement between Time 0 and Time 1 with the statement “*The best therapy for many people with mental illness is to be part of a normal community*” tends to significance (*p* = .07), showing that, in total, the dimension of “*Integration*” has evolved for many items.

After the training, the respondents were also less likely to agree with negative statements related to the fear and exclusion of mentally ill persons. The statements where the reduction was significant or very significant within the theme « *Fear and exclusion towards mental illness* », were the following:

- “*I would not want to live next door to someone who has been mentally ill”* (*p* = .002)*,*
- *“A woman would be foolish to marry a man who has suffered from mental illness, even though he seems fully recovered* » (*p* = .015).

Also, the reduction of agreement with the statement “*Locating mental health facilities in a residential area downgrades the neighborhood*” is very close to significance (*p* = .052).

Regarding the understanding of stigmatization, we noted in particular a very significant decrease in mean scores, meaning actually an increase (scoring has been reversed) of agreement with the statements saying “*We need to adopt a far more tolerant attitude toward people with mental illness in our society*” (*p* = .000).

Regarding the « *Causes of mental illness and needs for special services* », there was a very significant decrease (*p* = .004) in the notion of self-generated cause for mental illness. Thus, after the training, stigma mean score of the respondents decreased significantly on the statement “*One of the main causes of mental illness is a lack of self-discipline and will-power*”.

No significant differences were found relating to the reduction of stigma as a function of attendees’ gender and type of trainees (Initial Training versus Continuing Education).

## Discussion

The present study showed a lower level of agreement with stigmatizing statements, or a higher level of agreement with tolerant statements, as compared with other studies using the same instrument [17, 21]. In particular, most of the “*Integration*” and “*Fear and exclusion*” statements showed a significant reduction of stigma, suggesting an improvement in the perception of dangerousness and disturbance attached to persons with mental illness, a growing sense of integration in day-to-day life (neighborhood, marriage, employment), a shift towards the view that mental illness is similar to medical illness - a “*mental illness is like any other illness*” attitude -, and the expression of needs for ambulatory treatments and community-based therapies. Some statements of the latter two dimensions have also shown a reduction of stigma that almost reaches significance.

According to the literature, these findings can be linked with the higher education of our sample of students and professionals. Indeed, studies have generally shown that people with higher education and higher socio-economic status have more positive attitudes toward mentally ill people than the general population [22].

The status of health administrators or students of the respondents in our sample might also explain some differences in terms of the perceived level of services required for the mentally ill people. However, this is difficult to analyze in the present study, as Health Administrators in Continuing Education represent a limited proportion of the sample. That being said, similarly to other studies on contact-based education designed for health professionals [11, 13], our study highlights the importance of actual testimonies of consumers. Medical students and health professionals are described in the literature in this field as comparable to the general population, in their opinions about mental illness, even if the findings in this area have sometimes been controversial. Indeed, even if they have more knowledge and accept less restrictions toward mentally ill people, health professionals have as many negative stereotypes and the same social distance towards persons with mental illness, especially schizophrenia, as the general public [23]. A large review on the complex relationship between stigma and mental health professionals has shown that the latter can be stigmatizers, agents of destigmatization, as well as stigma recipients [24].

No significant differences between genders and types of trainees (Initial Training versus Continuing Education) were found, and this is in contrast with existing findings in, for example, the England *Attitudes to Mental Illness Research Report* findings, and other studies in which increased age, female gender and a high education level have also been associated with more tolerance and more positive attitudes [25, 26]. This could be explained by the small sample size of the present study and the small percentage of men and persons in Continuing Education.

The reduction of stigma measured after the short training intervention related to statements that were for the most regrouped in the themes “*Integration*” and “*Fear and exclusion*”. Interestingly, greater integration and less exclusion were also significant positive evolutions identified by the longitudinal analysis of the England *Attitudes to Mental Illness* survey between 1994 and 2012. This could mean that the statements about integration, fear and exclusion (or its counterpart, acceptance) are the most susceptible to change. Further research would be needed to explore these results.

In the present study, one must note that some items revealed at baseline a relatively low level of acceptance with negative and stigmatizing statements. More specifically, most of the 10 statements with the lower level of acceptance of stigmatization, visible at the end of Table 1 (which is organized following a decreasing order of baseline value), have not known significant changes between Time 0 and Time 1. This might be explained by the fact that there was little possibility to reduce stigma more on these particular subjects. For instance, the very low level of agreement with the exclusion of people with mental illness from public office may be associated with the fact that the French law has been imposing quotas of employment of handicapped people, among which mentally handicapped people, for decades, after the Law of July 10^th^ 1987.

An important difference between our results and those from the longitudinal analysis in England is that the statement “*One of the main causes of mental illness is a lack of self-discipline and will-power*” presents a very significant (*p* = .004) decrease of agreement after training, whereas in the England longitudinal analysis, no significant change was noticed over time. This may suggest that targeted training interventions explaining the multiple causes of mental illness could have an effect on representations about mental illness and associated stigma.

Researches aiming to evaluate the effect of targeted interventions on attitudes towards mental illness and using comparable measures have already taken place in the past. For example, in a previous study, a fieldwork placement of 74 Australian students working with people with mental illness in community settings resulted in more positive attitudes after completion [22]. This study used the *Community Attitudes towards the Mentally Ill* (CAMI) instrument [15], whose 40 statements are at the origin of the *Attitudes to Mental Illness* scale. The fieldwork placement had students spend one morning per week for six weeks in the community, helping consumers from community mental health centers, or running groups in the same community, with activities chosen by the consumers. In this study, contact with the mentally ill was shown to be a powerful tool in the education of future mental health professionals. It also lead to the development of more positive attitudes. In particular, after working with mentally ill people, the trainees did not perceive them to be violent nor dangerous. Finally, contact with mentally ill people seems a more powerful way of teaching students about mental health and changing their attitudes towards the mentally ill than are lectures or tutorials given by mental health professionals [22]. This finding could support the fact that the targeted training intervention that we used in our study, strongly based on users testimonies, lead to significant decrease in respondents’ perception of the dangerousness of mentally ill people.

However, another research using the *Community Attitudes toward the Mentally ill* (CAMI) scale has shown that targeted interventions efficiency is limited by stigmatizing media messages, which have a stronger effect. In one study in the U.S., 38 students viewed a fiction film depicting mentally ill people as dangerous and an information trailer reminding them that violence is not characteristic of mentally ill persons, 48 students viewed only the fiction film, and 19 students viewed a control fiction film that was not related to mental illness [27]. The CAMI scale was completed after the viewing. Those who saw the film linking mental illness and violence expressed significantly less favorable attitudes towards mental illness and community care of mentally ill persons than did those who saw the control film, regardless of whether or not they received the corrective information trailer. These results suggest that mass media stigmatizing portrayals of mentally ill persons have the power to counteract targeted interventions. It would then be interesting to include, in analyses of the results of targeted interventions aiming to reduce stigma about mentally ill people, an analysis of the background of mainstream representations in the media at the time of study [27].

A recent study, the first population-based survey in France of its kind, asked by Internet 1000 French persons about their current awareness, knowledge and attitudes towards mental illness [9] and established that negative views about mentally ill people expressed about a mental disorder per se decrease when linked to a specific individual. It also indicated a marked contrast between views of schizophrenia, compared with other disorders such as autism or bipolar disorder, with negative stereotypes of schizophrenia prevailing. Finally, while respondents quoted the media as their main source of information regarding mental disorders, the authors linked these results with the worrisome media portrayals of schizophrenia, that still have a powerful influence [9].

Future initiatives to challenge stigma and discrimination should consider disorder-specific initiatives rather than general approaches to mental illnesses, and use personal testimonies and raise the visibility of individuals with mental disorders living in the community, rather than producing generic campaigns attempting to de-stigmatize disorders [9]. These findings suggest that initiatives of this kind hold promise.

Several limitations should be noted, especially related to the specificity and size of our sample, and to the lack of control group. This latest limitation, especially, could be significant, as other causes, independent from our study, could explain the observed changes (for example news related to mental illness in the media, or broadcasting of a movie on this subject during the same period, etc.). Another limitation lies in the significant drop-out between Time 0 and Time 1 (with less than half of the respondents remaining at Time 1), which could induce a bias linked to a selection effect.

Also, the fact that our Time 1 was assessed within a month after the intervention does not allow us to evaluate the long-term duration of the short intervention effect. Indeed, there have been some concerns in the literature about the duration over time of anti-stigma interventions [10]. However, it may be that an in-person meeting with a psychiatric services user, with the emotions expressed during these personal testimonies, has more chances of being memorable than a generic media campaign.

Regarding the suitability of the scale that we have used for measuring a reduction of stigma, an important limitation is that it is not a stigma scale per se, but more a measure of the attitudes towards mentally ill people and mental illness. The scale is however considered as a good measure to track changes in attitudes towards mental illness, and is regularly used to evaluate the effect of anti-discrimination campaigns in the U.K. In the objective of using it as a measure for the reduction of stigma in a pre – post study, we have reversed the scoring of 14 items, so that every item looked negative and stigmatizing, a method which might offer some bias. Also, we performed a factor analysis, with a Varimax rotation method, and although the factors found in our analysis correspond to the dimensions of the original scale, a few differences were identified in some rare items. This could probably be explained by the small size of our sample and the fact that it is mostly constituted in women and educated people with prior training in the health sector, whereas the original English sample is very large and representative of the general sample. Finally, when using this measure, social desirability bias should be acknowledged as well.

Further research would therefore be needed to confirm our results, on a less specific and larger sample, and with a control group. Nevertheless, the methodology and the instrument used seem promising, in the context of the measurement of attitudes towards mental illness in France.

## Conclusion

The main interest of our study is to explore the potential efficiency of initiatives like a short intervention in reducing the stigma about mentally ill people among health administrators and students. This seems especially important knowing that, during their career, these professionals and future professionals could play a strategic role in mental health care and towards system users suffering from mental illness. More specifically, this research study suggests, like prior research conducted in other countries, the likely adequacy of targeted interventions, which would provide not only knowledge about mental illness to attendants, but also an opportunity for direct contact with mentally ill persons.

## Additional file

Additional file 1: The French translation of the attitude to mental illness scale. (DOCX 15 kb)

## Abbreviation

EHESP: Ecole des Hautes Etudes en Santé Publique
