# Supplementary material for: Could a short training intervention modify opinions about mental illness? A case study on French health professionals
Source: BMC Psychiatry. 2017 Apr 8;17:133. doi: 10.1186/s12888-017-1296-0 (PMC5385095; doi:10.1186/s12888-017-1296-0)
Supplement: Additional file 1: — The French translation of the attitude to mental illness scale. (DOCX 15 kb) [file 12888_2017_1296_MOESM1_ESM.docx]

French translation of the Attitudes to Mental Illness scale

*« Voici quelques opinions que certaines personnes ont à propos des personnes souffrant de troubles mentaux. Précisez s’il vous plaît dans quelle mesure vous êtes d’accord ou pas avec chacune d’entre elles, en cochant pour chaque proposition la case correspondante sur l'échelle suivante :*

- *Tout à fait d'accord*
- *D’accord*
- *Ni en accord ni en désaccord*
- *Plutôt pas d'accord*
- *Pas du tout d’accord*

1. L’une des principales causes de troubles mentaux est le manque de discipline de soi et de volonté
2. Les personnes souffrant de troubles mentaux dégagent quelque chose qui les rend facilement repérables par rapport aux personnes normales
3. Dès qu’une personne présente des signes de troubles mentaux, elle doit être hospitalisée
4. Les maladies mentales sont des maladies comme les autres
5. On devrait accorder moins d'importance au fait de protéger le grand public des personnes souffrant de troubles mentaux
6. Les hôpitaux psychiatriques sont un moyen dépassé de prise en charge des personnes souffrant de troubles mentaux
7. Tout le monde ou presque peut un jour souffrir de troubles mentaux
8. Les personnes souffrant de troubles mentaux sont l’objet de moqueries depuis trop longtemps
9. Nous devons adopter une attitude beaucoup plus tolérante à l’égard des personnes souffrant de troubles mentaux dans notre société
10. Il est de notre responsabilité de prendre en charge le mieux possible les personnes souffrant de troubles mentaux
11. Les personnes souffrant de troubles mentaux ne méritent pas notre compassion
12. Les personnes souffrant de troubles mentaux sont un fardeau pour la société
13. Augmenter les dépenses en faveur des services de santé mentale est un gaspillage d’argent
14. Il existe déjà suffisamment de services en faveur des personnes souffrant de troubles mentaux
15. On ne doit pas confier de responsabilités aux personnes souffrant de troubles mentaux
16. Il serait risqué pour une femme d'épouser un homme qui a souffert de troubles mentaux, même s’il semble parfaitement guéri
17. Je n’aimerais pas être le / la voisin (e) d’une personne qui a eu des troubles mentaux
18. Une personne ayant souffert de troubles mentaux ne doit pas pouvoir accéder à un emploi ou à un mandat public
19. Personne n’a le droit d’exclure les personnes souffrant de troubles mentaux de leur quartier
20. Les personnes souffrant de troubles mentaux sont bien moins dangereuses que ces que pensent la plupart des gens
21. La plupart des femmes ayant déjà été hospitalisées en psychiatrie peuvent être embauchées comme babysitters en toute confiance
22. La meilleure thérapie pour de nombreuses personnes souffrant de troubles mentaux est d’être intégrées au sein d’une communauté normale
23. Dans la mesure du possible, les services de santé mentale doivent être délivrés dans le cadre de structures de proximité
24. Les résidents n’ont rien à craindre des personnes qui viendraient dans leur quartier pour bénéficier de services de santé mentale
25. L’idée que des personnes souffrant de troubles mentaux puissent vivre dans des quartiers résidentiels fait peur
26. Le fait d’ouvrir des structures psychiatriques dans une zone résidentielle dévalorise le quartier
27. Les personnes souffrant de troubles mentaux doivent avoir les mêmes droits que les autres en matière d’emploi »
